# Dentistry a Specialty in Medicine

**Published:** 1888-08

**Authors:** 


### Editorial.

### DENTISTRY A SPECIALTY IN MEDICINE.

No one at the present day questions the position of dentistry as a branch of the healing art, and as such a specialty in medicine. The open question, however, remains as to the manner in which recognition shall be extended to us by the medical profession, whether as a body or as individuals. The individual members of the profession who have done most to secure recognition for the body corporate have been liberally or medically educated men. It cannot be denied but that it was through the efforts and personal standing of these men that the profession was seated as a body in the American Medical Association and the Ninth International Congress. Yet no restriction was placed upon any one. The door was opened to all to unite with the medical profession, and form a section in their annual meetings, whether they possessed an M. D. or not. By its action the American Medical Association plainly said that it was willing and glad to recognize us as specialists in medicine, and by so doing it came fully half way to meet us; overlooking our deficiencies, it accorded us a hearty welcome. To turn our backs upon them now would indeed be unappreciative, to say the least. Those who have been most strenuous in their demands for separate professional standing have been entirely disarmed by the open recognition accorded. Their principal argument has been that the medical profession would not recognize us, hence we should stand upon our dignity and form a separate profession. That ground has been entirely removed, and full and fair recognition been accorded. In view of our many well-known shortcomings we should accept the recognition extended, and try to merit the confidence reposed in us by immediately advancing the standard of dental education, and in every way possible elevating the position of our degree.

There is a vast difference between practicing medicine and practicing a specialty *in* medicine. The first involves a practical knowledge of the art of medicine, while the latter requires only a general knowledge of the science and a special knowledge of the art of the branch elected to pursue. It is not necessary that an individual, in order to be recognized as a medical specialist before the law, should possess a medical degree. All that the law requires is that he obtain a certificate of proficiency in medicine from the State Board of Examiners in the State where he desires to practice. Many of the graduates of our dental colleges are sufficiently well prepared to pass the required examination. All that would be necessary would be for them to spend a short time on those few branches that are not specially required in the dental curriculum, notably the science of practice and obstetrics. Anatomy, chemistry, surgery and physiology are the same for both schools. Why any one should claim that because a student elects to substitute the art of dentistry for the art of medicine and obstetrics he should be debarred from recognition as a specialist in medicine is beyond comprehension. Just so soon as the branches common to medicine and dentistry shall be taught equally well in dental as in medical colleges, then will the distinction be done away with. But even then, unless we perfect ourselves in the art as well as the science of medicine, we should not have the right to receive the title of M. D. That title should be reserved for fully qualified graduates in medicine, and should not be conferred upon dental graduates. Because the law gives us the right to practice a branch of the healing art, that is no reason why we should assume to practice the whole. The American Medical Association did not elect to issue certificates of proficiency in obstetrics to those members of the dental profession who availed themselves of the privilege offered to form a dental section in their body, but it did say it recognized us as their protégé because all that we have that has elevated us above a trade has been derived from medical science. Step by step, one after another of the medical branches have been incorporated into the dental curriculum, until now it is considered essential to employ largely medically educated men to instruct in those branches. We have been most magnanimously recognized as specialists in medicine by the medical profession; such recognition does not, however, establish us as such in the eyes of the law. Before such a standing shall be attained we must incorporate the science of medicine and obstetrics in our curriculum, and require proficiency in these branches before granting the degree of D. D. S. When this is done, and not before, will immunity from legal prosecution for ill results of practice that does not strictly belong to our specialty be secured. This question is one of very considerable interest to us as a profession. Those who are practicing implantation, having none other than the title of D. D. S., or not having qualified before the law, are laying themselves liable to legal prosecution for every case in which they operate, and their position would not be a very agreeable one in case of bad results.

